# Observer-rated outcomes of communication-centered treatment for adults who stutter: A social validation study

**DOI:** 10.1371/journal.pone.0303024

**Published:** 2024-05-16

**Authors:** Courtney T. Byrd, Geoffrey A. Coalson, Danielle Werle

**Affiliations:** 1 Arthur M. Blank Center for Stuttering Education and Research (AMBCSER), The University of Texas at Austin, Austin, Texas, United States of America; 2 AMBCSER, Atlanta Satellite, Atlanta, Georgia, United States of America; Utah State University, UNITED STATES

## Abstract

Previous studies have reported that adults who stutter demonstrate significant gains in communication competence, per self-ratings and clinician-ratings, upon completion of a communication-centered treatment, or CCT. The purpose of this social validation study was to determine whether communication competence ratings reported by untrained observers are consistent with client and clinician judgments of communication competence gains following CCT. Eighty-one untrained observers completed an online survey that required each to view one of two videos depicting an adult who stutters during a mock interview recorded prior to CCT or after CCT. Observers were then asked to rate the communication competence of the interviewee on a 100-point visual analog scale and provide additional demographic information. Communication competence of the adult who stutters who had completed CCT was rated significantly higher in their post-treatment video. Upon controlling for two demographic factors found to be associated with observer ratings (years of education, years the observers had known an adult who stutters), significantly higher ratings of communication competence for the post-treatment video were maintained. These preliminary findings provide social validity for CCT by demonstrating that the gains in communication competence reported in previous studies through clinician and client observations are also reported by untrained observers who are not familiar with CCT.

## Introduction

Public perception that adults who stutter are poor communicators is pervasive. Decades of research illustrate the widespread belief that competent communication–a skill that is considered essential for academic success (e.g., [1]), workplace advancement (e.g., [2–5]), and interpersonal relationships (see [6,7])–cannot be adequately attained in the presence of stuttered speech. Based on this assumption, treatment options for adults who stutter have historically focused, in part or whole, on learning to speak fluently, and/or modifying moments of stuttered speech (see systematic review by Brignell et al. [8]). Although clinical trials are available that indicate post-treatment fluency gains are achievable for some adults (e.g., [9–11]), these reviews also note considerable individual variability. Only recently have clinical researchers demonstrated that targeting fluency during treatment is not necessary to improve the communication competency or quality of life of persons who stutter [e.g., 12–14]. These findings align with contemporary views of stuttering, which are anti-ableist in nature, and do not view stuttering as a condition that needs to be fixed (e.g., [12–14]).

Byrd and colleagues [15–17], for example, explored the impact of participation in a *communication-centered treatment (CCT)* designed to improve communication competence with no attempt to change speech fluency. Participants were rated to be significantly stronger communicators post-treatment by clinicians who were unfamiliar with the participants and blinded to pre-/post-treatment status of video samples [15,16]. Participants themselves also reported significantly stronger communication competencies after treatment and across a variety of speaking contexts (dyad, small group, large group, public presentation) and listeners (strangers, acquaintances, friends; Coalson et al. [17]). Neither clinician- nor self-ratings of communication competency were predicted by pre-treatment stuttering frequency [15,16] or changes in stuttering pre- to post-treatment [17].

Although CCT appears to provide meaningful outcomes from the perspective of clinicians and participants who have received this treatment, ratings from the general public (i.e., observers unfamiliar to CCT) are needed to provide social validation (e.g., [18,19]). Schloss and colleagues [20] examined the social validity of clinician-reported outcomes of a treatment designed to increase assertiveness during communication exchanges of three adults who stutter. Ten graduate students naïve to the treatment randomly viewed one of two videos, either pre-treatment or post-treatment, and rated the speaker’s assertiveness during the interview. Based on higher assertiveness ratings from the naïve observers for post-treatment video samples, researchers concluded that the potential treatment effects could be extended to the general public. A second social validation study by Schloss and colleagues [21, *n* = 11 naïve observers, *n* = 3 adults who stutter] replicated the previous outcomes with respect to observed assertiveness of the same three adults who stutter post-treatment. Interestingly, outcomes across these two studies also found that post-treatment changes in assertiveness demonstrated an inconsistent relationship with post-treatment stuttering, suggesting that changes in communication can be achieved independent of changes in fluency.

Taken together, the available clinical data for treatments that are communication rather than fluency centered provide preliminary but compelling evidence that fluency and communication are not inextricably linked. Specific to the data available regarding CCT, both the participants and the clinicians in the prior studies had shared knowledge of the nature of stuttering, the focus of CCT, and the desired clinical outcomes. That is, there was a relatively shared criteria for subjective evaluation in a controlled clinical setting. One could argue that these criteria may diverge from the appraisal used by unfamiliar, untrained laypersons. In addition, clinicians who participated in the previous CCT studies were blind to the pre- versus post-treatment status of videos, and videos were randomized, but they were evaluating a large number of consecutive videos depicting participants who stutter–a scenario rarely encountered in everyday life which may have potentially compromised their ratings. A social validation study, similar to Schloss et al. [20,21], would address these potential rating biases and provide confirmation whether observations made by clinicians, and prior participants, are similarly observed by the general public.

Therefore, to extend previous findings to a more socially valid context, the present study examined whether post-treatment gains in communication competency observed by clinicians and self-reported by participants in previous studies are also identifiable to untrained observers. To do so, we recruited a large cohort of untrained observers to rate the communication competency of an unfamiliar adult who stutters based on a video sample recorded either before treatment or after treatment. To explore implicit factors known or suspected to influence social evaluation, and evaluation of people who stutter (e.g., [22,23]) in particular, we also considered to what extent demographic and observer-related factors may account for perceived communication competency ratings for each video sample.

### Treatment for adults who stutter

Three systematic reviews [9–11] have found that positive treatment outcomes are associated with a remarkable variety of treatments and a variety of related clinical factors (see Johnson et al. [11] for comparison of client-based, intervention-based, and interpersonal-based factors in stuttering treatment outcomes). To date, the majority of treatment approaches for adults who stutter primarily or exclusively target fluency-centered speech techniques intended to either eliminate or minimize moments of stuttered speech (i.e., fluency shaping [24,25]; stuttering modification [26]). Studies have demonstrated that fluency gains following fluency centered treatment are observed, even 3- to 12- months post-treatment (e.g., [27–29]). Yet, from the few randomized control trials (RCTs) that exist for adults who stutter, it is evident that fluency-centered treatment (a) has an inconsistent impact on the psychological consequences of stuttering [28,29]), (b) is prone to high rates of relapse (71% [30]), and (c) may compromise the speaker’s innate ability to communicate (e.g., unnatural, effortful, and/or incongruent with their identity [31]). Additionally, listeners often rate speech techniques employed during treatment to achieve fluency as equally or less desirable than stuttered speech [32–34].

Furthermore, several recent studies indicate that stuttering severity does not predict communication attitudes in persons who stutter regardless of age (e.g., children [35]; adults [36]). These data suggest that the assumption that fluency must be targeted to facilitate positive perspectives of self and/or communication in persons who stutter may be misleading. In fact, Byrd and colleagues [15–17,37,38] provide evidence that significant positive changes in communication attitudes, and communication competence, can be reliably obtained through participation in a treatment that focuses on improving overall communication and explicitly excludes clinical goals that attempt to eliminate, or modify stuttered speech.

### Preliminary CCT outcomes with children who stutter

Byrd et al. [37, *n* = 23, ages 7- to 14-years old] examined changes in cognitive and affective wellbeing before and after treatment reported by 23 children and adolescents who stutter and their parents. Specifically, adolescents reported greater quality of life (as measured by the Overall Assessment of Speaker’s Experience with Stuttering [39]) following treatment, and parents reported significant improvement in their child’s ability to establish peer relationships (as measured by the PROMIS-Pediatric Short Form Peer Relationships Scale [40]). A follow-up study by Byrd et al. [38] replicated these findings in an additional 23 child and adolescent participants (ages 7- to 14-years old). That is, participants and their parents reported significant post-treatment gains in quality of life and peer relationships. Taken together, these findings suggest that treatment that excludes any attempt to modify speech fluency, and instead targets communication competencies, may result in significant gains that meet or exceed those previously reported for fluency-focused or stuttering modification treatment approaches.

Byrd et al. [15] extended analyses of their communication centered, whole person approach, by examining communication competencies in 37 children and adolescents who stutter (ages 4- to 17-years old) pre- versus post-treatment. An unfamiliar clinician rated pre- and post-treatment presentations (3 to 4 minutes in length), recorded in front of a large group of peers, based on nine different communication competencies: (1) language use, (2) language organization, (3) speech rate, (4) intonation, (5) volume, (6) gestures, (7) body position, (8) eye contact, and (9) facial affect (for detailed description, see Byrd et al. [15,16]). Findings provided preliminary evidence that, in addition to replicating the positive post-treatment changes in cognitive and affective aspects of stuttering reported in prior studies (Byrd et al. [37,38]), clinicians rated communication competency of children who stutter as significantly stronger during presentations recorded after treatment. Of particular relevance to the present study, these changes in communication competence following CCT were not significantly predicted by pre-treatment stuttering frequency.

### Preliminary CCT outcomes with adults who stutter

Positive post-treatment gains in communication competence after treatment for children who stutter have been replicated in adults who stutter who have also participated in CCT. Coalson et al. [17] examined self-reported clinical outcomes from 33 adults who stutter after an 11-week communication-centered treatment (for greater detail, see S1 Appendix) similar to the one-week treatment program for children described in Byrd et al. [15,16,37,38]). During the first and last week of treatment, participants completed the Self-Perceived Communication Competence [41]—a brief scale designed to self-assess communication competence in four specific communicative contexts (dyad, small group, large meeting, presentation) with three interlocutors (stranger, friend, acquaintance). Significant gains in self-rated communication competence were reported post-treatment and, similar to the children and adolescents who stutter in Byrd et al. [15], post-treatment gains were not predicted by stuttering frequency.

These self-reported outcomes by adults who have completed CCT have also been found through clinician observation. Byrd et al. [16] examined post-treatment communication competencies in 11 adults who stutter. Each participant completed a mock interview with an unfamiliar interviewer in the week prior to treatment, and in the week after completion of treatment. Randomized video samples of these pre-and post-interviews were rated offline by a certified and licensed speech-language pathologist unfamiliar with the speaker and blind to pre-/post-treatment status of each video. As with the clinician observations for children and adolescents who have participated in CCT [15], adults who stutter demonstrated observable post-treatment improvements in eight of the nine targeted communication competencies (i.e., language use, language organization, speech rate, intonation, volume, gestures, body position, eye contact, and facial affect), and again, these improvements were not predicted by pre-treatment stuttering frequency. Taken together with Coalson et al. [17], these preliminary data suggest that expert clinicians, as well as the adults who stutter themselves, observe positive changes in communication competencies after completing CCT that are independent of pre- and/or post-treatment stuttering.

### Untrained observers

Although the participant- and clinician-based outcome measures used in Byrd et al. [15–17] are commonplace within clinical trials of adult stuttering treatment (e.g., [28,42–45]; however, see [46–49] for third-party ratings of naturalness), it could be argued that the changes reported were evaluated from two parties–the participant and the clinician–whose shared perspectives invite a potential for rater bias. A logical means to address potential rater biases due to familiarity with the condition, and/or its treatment, is to examine clinical outcomes from the perspective of raters who have neither–the untrained observer.

To date, post-treatment communication competence of adults who have completed CCT has yet to be explored from the perspective of the untrained observer. Unlike clinicians or participants, who have shared understanding of communication competence in the context of CCT, untrained observers provide a valuable means to assess the social validity of any communicative outcome measure, by virtue of the inherently variable intrinsic and extrinsic cues that they may use to evaluate a speaker’s communication competence. By assessing the perspective of a large group of untrained observers, we can capture the variance of such internal criteria while also measuring the broader impact of CCT outcomes. Thus, our primary research question is to assess to what extent the gains in communicative competence observed by clinicians, and self-reported by participants, in previous studies are also evident to the general public. Given the potential influence of generalized biases towards stuttering, and persons who stutter, a secondary question is to what degree specific demographic factors may mediate the general public’s perception of CCT outcomes.

### Potential observer-based demographic mediators

Among the general public, there is a well-documented negative bias towards persons who stutter (e.g., [50–53]). A number of demographic factors have been found to influence an untrained observer’s evaluation of any speaker (e.g., age, gender, education, occupation, familiarity with language/multilingualism), including those who stutter (see [23,54,55]). Such demographic factors as well as additional observer-based factors have a potential influence on naïve observers’ attitudes towards adults who stutter (e.g., familiarity with a person who stutters [56,57]; personal history with a communication disorder [58,59]; visible and/or invisible disability [60]).

Additionally, factors known to mitigate an observer’s overall evaluation of an adult who stutters as a *person* may override any attempt to measure a targeted trait, such as communication competence, resulting in overly positive evaluations (see Werle & Byrd [61] for positive feedback bias by professors when evaluating presentations by students who stutter) or overly negative evaluations (see Byrd et al. [22], for gender bias towards adults who disclose stuttering). Thus, it is plausible that observer ratings of the communication competence of a particular adult who stutters may be driven entirely by their overall perception of all people who stutter. Therefore, in the present study we also aimed to explore and account for observer-based demographic factors that may mediate the ratings of unfamiliar observers.

### Rationale for the present study

In sum, the primary aim of the present study was to examine the social validity of post-treatment gains in communication competence of an adult who stutters who completed CCT—an approach to treatment that focuses on communication and makes no direct or indirect attempt to increase fluency or modify stuttered speech. A secondary aim of this study was to assess whether observer-based factors of untrained observers influence perceived communication competence.

**Research Question 1 [RQ1]:** Do untrained observers, similar to expert clinicians, perceive higher communication competence for an adult who stutters following CCT?

**Research Question 2 [RQ2]**: Do observer-based demographic factors predict evaluation of the communication competence of an adult who stutters?

## Methods

The following study was approved by the Institutional Review Board at the University of Texas at Austin (IRB: 2015-05-0044, [62]). Survey participants indicated consent prior to participation by clicking to advance to the first survey question after reading the cover letter that includes a description of the study, potential benefits and minimal risk of voluntary participation, and compensation for participation ($0.50 per participant, similar to Werle and Byrd [61,63,64]). Communication competency stimuli consisted of two separate videos: one depicting a speaker before he had completed CCT (Pre-treatment Video) and one depicting the same speaker after he had received CCT (Post-treatment Video). Written consent was obtained from the adult depicted in the video stimuli prior to his self-selected enrollment in CCT.

### Communication-centered treatment (CCT)

A detailed description of the treatment program is provided in Byrd et al. ([16], see also Coalson et al. [17] and Byrd [65]) and summarized in S1 Appendix. The overarching goals are to ensure individuals who stutter communicate competently, advocate for themselves in a manner that maintains agency, and ensure their quality of life does not depend on reducing, or attempting to control, stuttered speech. In brief, adult participants complete 11 weeks of treatment consisting of two 60-minute sessions per week (one group session, one individual session), totaling 22 sessions which include training in Communication, Advocacy, Resilience, and Education (the Blank Center CARE^™^ Model). With respect to Communication, participants receive explicit instruction on how to strengthen nine core communication competencies (i.e., language use, language organization, speech rate, intonation, volume, gestures, body position, eye contact, facial affect). All competencies are addressed via a pragmatic as opposed to a fluency framework. For example, as opposed to changing rate, volume, or tone to potentially facilitate fluency, participants learn the importance of changing rate, volume, and intonation with respect to speaking context (e.g., giving a presentation vs. having a one-on-one conversation), and how changes in these competencies can influence both the meaning of their message and listener engagement. Participants also learn that stuttering is an independent construct from communication competence, and that efforts to not stutter may compromise communication.

Training during individual sessions provided an opportunity for participants to review what would be covered in weekly group sessions, to prepare for the activities, and to debrief about topics covered in group sessions from the prior week. During group sessions, participants strengthened their communication competence across a number of distinct, functional yet challenging speaking scenarios, including mock job interviews, small group interactions, impromptu icebreakers, one-on-one interactions with unfamiliar persons, and multiple presentations varied both in purpose (e.g., informative, persuasive, inspirational) and audience composition (e.g., small and large groups, familiar and unfamiliar listeners).

### Communication competency stimuli

Two video samples (Pre-treatment Video, Post-treatment Video) from an adult who stutters who participated in Byrd et al. [16] and Coalson et al. [17] were selected for stimuli. Table 1 provides a summary of both video stimuli.

**Table 1:** Characteristics of pre-treatment and post-treatment video stimuli.

	Pre-treatment Video	Post-treatment Video
%SS^a^	10.60%	8.24%
Total words	453	959
SSI-4^b^	Moderate	Moderate
Frequency	7	6
Duration	10	10
Physical Concomitants	10	9
Total	27	25
Observer-Rated Severity (100-point VAS)		
*M* (SE)	64.71 (2.54)	64.95 (2.41)
*N*	63	57
Length of Video	5 min, 30 sec	6 min, 25 sec

^a^Percent of stuttered speech, disfluency types based on Yairi and Ambrose [66].

^b^Stuttering Severity Instrument-4^th^ Edition [67].

Each of the two video stimuli depicted a one-on-one, in-person mock interview between (a) the same adult who stutters, who served as the interviewee, and (b) a clinical staff member unfamiliar to the interviewee who served as the interviewer. Interviewers differed between pre- and post-treatment. Each interviewer was unfamiliar with the participant who served as the interviewee, and both were provided identical, commonplace interview questions as prompts (e.g., “What do you consider your strengths and weaknesses?”; “Describe a prior work-related issue and how you addressed it.”).

Selection criteria for video stimuli included (a) significant intra-speaker gains in communication competence as rated by the speech-language pathologist evaluator, and (b) comparable stuttering frequency pre- to post-treatment. The Pre- and Post-treatment Video samples used in the present study were as close to the same percentage and severity possible, though not identical given the natural variability in stuttering frequency and sample length. As detailed in Table 1, stuttering frequency was 10.6% for the Pre-treatment Video and 8.2% the Post-treatment Video, and stuttering severity for both the Pre- and Post-treatment Videos were rated as *moderate* per the Stuttering Severity Index– 4^th^ Edition (SSI-4 [67]). To confirm there were no perceptible differences in stuttering between the two video samples, a validation survey (administered as part of a separate project, Byrd et al. [68]) asked a separate cohort of untrained observers not included in this study to rate stuttering severity using a 100-point visual analog scale (0 = no stuttering, 100 = extremely severe stuttering) after rating one of the two video samples. Untrained observers rated stuttering severity to be statistically comparable (*p* = .95) between Pre- and Post-treatment Video samples, with nearly identical mean severity ratings (*M* = 64.71 and 64.95, respectively). The participant who completed the CCT for which his pre-treatment and his post-treatment interview served as the stimuli for the present study was a 19-year-old Hispanic male who stutters. The participant self-identified as a monolingual English speaker, with no prior or present communication, developmental, psychological, neurological, and/or physical concerns.

### Survey administration and observer description

The Pre- and Post-treatment Video samples were embedded in a Qualtrics-based survey distributed to adult untrained observers, with the survey prompting one of the two videos in succession of access to ensure a random yet comparable number of participants observed one of each of the two samples. Potential untrained observers were recruited using the crowdsourcing platform Amazon Mechanical Turk (MTurk) which allows a large and diverse pool of individuals to complete surveys for compensation ($0.50) after requirements for quality were met (e.g., attention/comprehension checks [69]). This amount was determined based on pilot research for previous studies [61,63,64] that assessed engagement and quality of responses. No filters were applied to the recruitment of untrained observers other than location (USA) and respondent approval rate greater than 95% (i.e., rate in which respondents completed surveys were accepted by researchers). Occupations were self-reported by respondents. Unlike previous social validation studies by Schloss et al. [20,21] that recruited college students enrolled in coursework related to communication disorders or special education, the untrained observers in the present study were older (*M* = 42.4 years) and relatively few self-reported occupations in allied health (*n* = 9 observers [11.11%]; Pre-treatment Video, *n* = 4, Post-treatment Video, *n* = 5) or education (*n* = 6 observers [7.41%]; Pre-treatment Video, *n* = 4, Post-treatment Video, *n* = 2).

Each survey began with an informed consent landing page, followed by the instructions: “You are about to watch a video of an interview. Immediately following the video, you will be asked questions about the interviewee. The interview will be approximately 5 to 7 minutes in length. You will only be able to move forward in the survey after you have watched the video in its entirety.” The untrained observer then watched a single video of the participant—either the Pre-treatment Video or the Post-treatment Video—with the advance button disabled for both the survey portal and the embedded video. Immediately after their viewing of the video, the untrained observers were provided the following instructions accompanied by a 0–100 visual analog rating scale: “Using the scale below, please rate the interviewee’s communication skills. 0 = Communication skills not at all effective, 50 = Communication skills somewhat effective, 100 = Communication skills extremely effective.” The term ‘communication’ was not operationally defined for the observers as allowing the definition of communication to freely vary among observers maintained social validity of data. Upon providing their rating, participants were then asked to describe what factors led to their rating in a free response text box. The subsequent free-text response provided an opportunity to further explore what criteria observers used to rate communication competence without a priori suggestion by the researchers (see S2 Appendix for qualitative analysis of free response data for each video sample).

Following their rating and subsequent free response, observers were asked to provide demographic information (e.g., age, race, ethnicity, gender, education, occupation, primary language). Observers were also prompted to report their personal relationship with stuttering, persons who stutter, or other communication differences (i.e., “Are you a person who stutters? Do you personally know a person who stutters? If so, please describe your relationship and how long you have known this person. Have you had previous speech, language, and/or hearing evaluation or therapy?”) as well as any visible or invisible diagnoses unrelated to communication difficulties (i.e., physical condition, psychological condition, neurological condition, emotional condition, vision/hearing loss, reading condition, other/describe, none). Each survey included three attention check questions and four comprehension check questions to assess the integrity of individual responses. Survey data were collected in July 2021, and all observers were paid in accordance per MTurk standards of distribution.

The survey was initiated by 128 potential observers (67 Pre-treatment Video, 61 Post-treatment Video). Of these 128 potential observers, 36 (19 Pre-treatment Video, 17 Post-treatment Video) did not pass at least one of the seven attention/comprehension check questions during the course of the survey and were excluded from final analysis. Of the remaining 92, four were excluded because they self-identified as a person who stutters (2 Pre-treatment Video, 2 Post-treatment Video). Seven additional observers were excluded (3 Pre-treatment Video, 4 Post-treatment Video) due to free-response items that suggested unclear understanding of the task (e.g., “*She [the interviewer] can ask some more questions*.”), questionable attention to the study (e.g., “*Everything is perfect*.”), or potentially auto-generated responses (e.g., unusual format of free-responses repeated across items or participants). The final corpus included 81 observers (43 Pre-Treatment Video, 38 Post-Treatment Video). See Table 2 for detailed description of demographics.

**Table 2:** Demographic characteristics of untrained observers.

	Video stimuli		
	Pre-treatment	Post-treatment	*N*
*N*	43	38	81
Age	43.4 (16.4)	41.8 (16.5)	42.4 (16.5)
Race			
Native American or Alaskan Native	0	0	0
Asian	8	5	13
Black or African American	6	3	9
Native Hawaiian or Pacific Islander	0	0	0
White	27	29	56
Other Identification	2	1	3
Ethnicity			
Not Hispanic or Latino	38	31	69
Hispanic or Latino	5	7	12
Self-Identified Gender			
Male	19	24	43
Female	24	14	38
Other Identification	0	0	0
Years of Education	16.4 (2.7)	16.5 (3.0)	16.5 (2.8)
Primary Language			
Bengali	0	1	1
English	36	31	67
Filipino	1	0	1
French	0	1	1
Hindi	0	1	1
Marathi	1	0	1
Portuguese	2	1	3
Saurashtra	1	0	1
Swedish	0	1	1
Tamil	2	1	3
Turkish	0	1	1
Knows adult who stutters	25	19	44
Years known	12.6 (17.0)	9.7 (14.2)	11.2 (15.8)
Invisible or mixed disability	8	8	16

*Note*. Means and standard deviations (in parenthesis) reported for age, years of education, and years known.

### Analyses

#### RQ1: Do untrained observers, similar to expert clinicians, perceive higher communication competence for an adult who stutters following CCT?

An independent *t*-test was conducted to compare untrained observers’ evaluation of communication competency depicted in one of two stimuli—either the Pre-treatment or the Post-treatment Video of an adult who stutters who received CCT. Video time point (Pre-treatment Video, Post-treatment Video) served as the independent variable, and ratings from the 100-point visual analog scale (VAS) of communication competence served as the dependent variable (0 = low competence, 100 = high competence). The independent *t*-test was two-tailed (α = .05) and effect sizes were calculated and interpreted using Cohen’s *d* [70]. Because of the preliminary nature of the data and modest sample size, findings were verified by non-parametric analysis (Mann-Whitney *U*, α = .05).

#### RQ2: Do observer-based demographic factors predict evaluation of the communication competence of an adult who stutters?

A linear regression was conducted to assess the influence of viewing communication competency stimuli (Pre-Treatment Video, Post-treatment Video) and nine observer-related variables (i.e., age, race, ethnicity, gender, years of education, non-English primary language, knowing an adult who stutters, number of years the observer has known an adult who stutters, invisible diagnosis; see Table 2) upon ratings of the communication competence of an adult who stutters. Categorical variables with responses that were either not reported (i.e., non-binary self-identified gender) or reported infrequently (i.e., non-English primary language with fewer than 4 observers) were transformed to create a single binary variable (i.e., male/female; English/non-English primary language). In addition, to maintain relatively even distribution amongst categories during analysis, race had to be analyzed as a binary variable due to relatively infrequent self-identification as Asian (*n* = 13, Pre-treatment Video = 8, Post-treatment Video = 5), Black/African American (*n* = 9; Pre-treatment Video = 6, Post-treatment Video = 3) or racial identification that was not included in existing categories (*n* = 3; Pre-treatment Video = 2, Post-treatment Video = 1).

To determine which of the nine observer-related factors held meaningful predictive value of observer ratings, and therefore qualify for entry into the linear regression, we applied a version of Hosmer et al.’s [71] step-by-step method for *purposeful selection of covariates* modified for OLS linear regression. First, nine univariate analyses were conducted for each variable (chi-square tests for categorical variables, independent t-tests for continuous variables). Variables with *p*-values greater than 0.25 were excluded. Second, a model with non-excluded variables was fitted, then each predictor re-assessed and deleted if significance exceeded *p* > .05. Third, the reduced model was compared to the original model using *F* values to ensure improved fit and to verify that change in beta coefficients between models did not exceed 20% (i.e., deleting-refitting-verifying cycle). Fourth, any variables that were excluded during the initial step were re-entered into the model, one at a time, but retained only if *p*-values were less than .05. Fifth, any interaction terms of interest between the remaining variables were entered into the model. Interaction terms were assessed using the deleting-refitting-verifying cycle used for main effects and retained only if statistically significant at *p* < .05 and if model fitness improved. Any main effects and interaction terms remaining after these steps were completed comprised the final model (see Table 3). Due to the preliminary nature of the data and modest sample size, bootstrap analysis was conducted to confirm initial findings (95% confidence intervals; 5000 samples). Effect sizes for individual predictors within linear regression were calculated and interpreted using *f*^*2*^ (.02 = small, .15 = medium, .35 large [70]).

**Table 3:** Summary of regression analyses of stimuli (pre-treatment video, post-treatment video) and observer-based factors predicting communication competence of an adult who stutters, as rated by untrained observers.

	Variable	*B*	95% CI	*β*	*t*	*p*	*F*	*df*	*R* ^*2*^
Model 1	Intercept	-5.25	-11.34, .78		-1.68	.089	6.03	1, 79	.071
	**Pre-/Post-treatment Video**	**11.24**	**2.38, 20.11**	**.27**	**2.46**	**.013**			
Model 2	Intercept	-5.68	-11.25, -.11		-1.95	.046	7.30	3, 77	.221
	**Pre-/Post-treatment Video**	**12.12**	**3.97, 20.26**	.29	2.84	**.004**			
	Years of education	-2.47	-3.92, -1.03	-.33	-3.28	< .001			
	Years AWS known	.23	-.03, .49	.17	1.67	.087			
Bootstrapped	Intercept		-11.59, .07			.063			
	**Pre-/Post-treatment Video**		**3.88, 20.72**			**.007**			
	Years of education		-3.99, -.85			.003			
	Years AWS known		.01, .46			.049			

*Note*. CI = confidence interval for unstandardized beta coefficients; AWS = adult who stutters.

## Results

**RQ1**: **Do untrained observers, similar to expert clinicians, perceive higher communication competence for an adult who stutters following CCT?**

**Fig 1:**
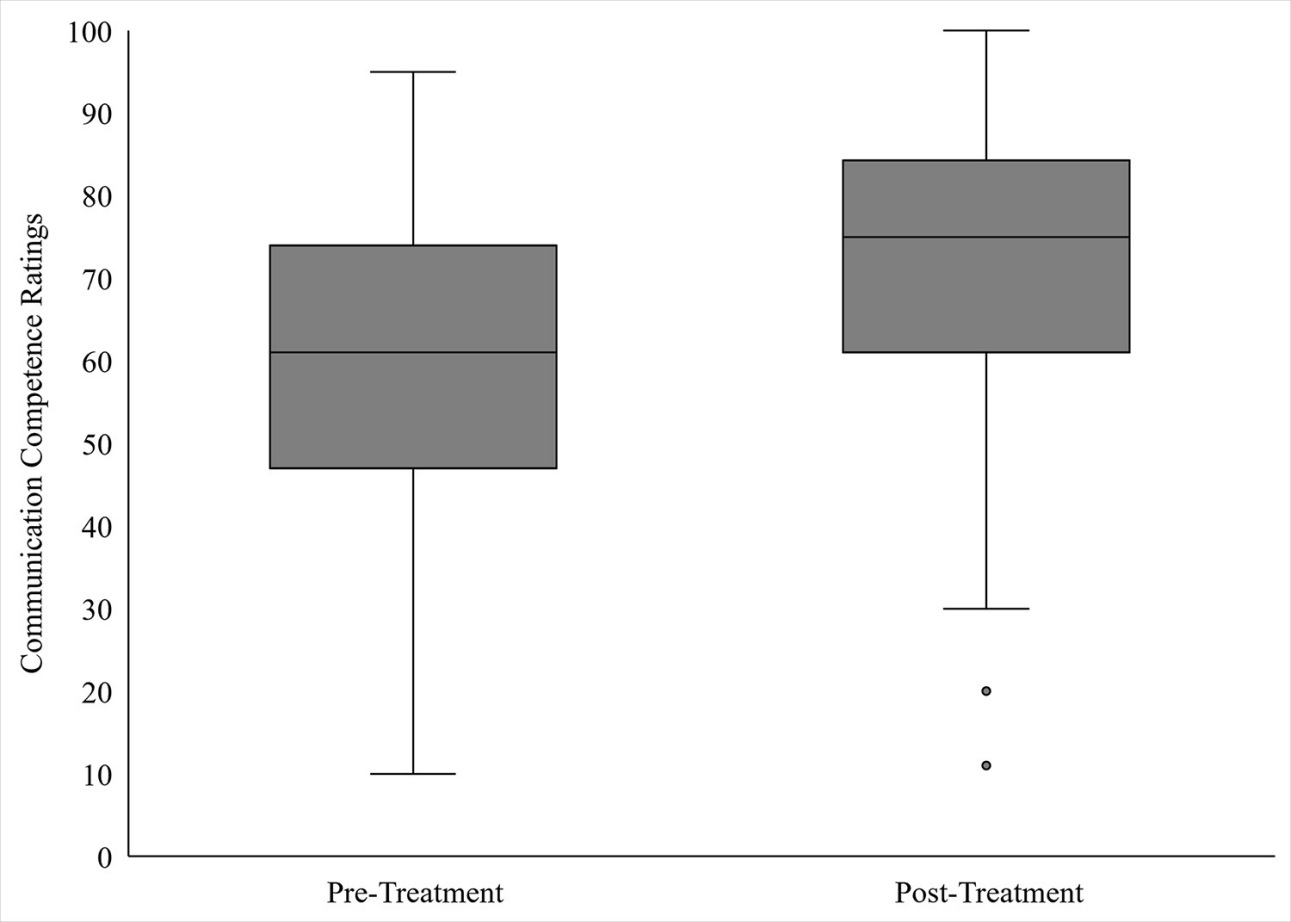
Communication competence of an adult who stutters, as rated by untrained observers before CCT (pre-treatment video) and after CCT (post-treatment video).

An independent *t*-test was conducted to assess how untrained observers rate communication competence of an adult who stutters. As depicted in Fig 1, findings reveal significantly stronger perceived communication competence for the Post-treatment Video (*M* = 70.3, *SD* = 21.1) than the Pre-treatment Video (*M* = 59.0, *SD* = 20.1), *t*(79) = 2.46, *p* = .016, *d* = .55 [medium effect size]. Findings were confirmed via nonparametric analysis, *U*(43,38) = 532.50, *z* = 2.70, *p* = .007.

**RQ2**: **Do observer-based demographic factors predict evaluation of the communication competence of an adult who stutters?**

A linear regression was conducted to determine the contribution of nine observer-related factors (i.e., age, race, ethnicity, gender, years of education, non-English primary language, knowing an adult who stutters, number of years observer has known adult who stutters, visible or invisible diagnosis) upon ratings of the communication competence of an adult who stutters. As expected, the Post-treatment Video was a significant predictor of higher ratings of communication competence when entered as the lone predictor variable (β = .27, *p* = .013), explaining 7.1% of the variance (*R*^2^ = .071; *F*(1, 79) = 6.03, *p* = .016; see Model 1 in Table 3). Upon completing Hosmer et al.’s (2013) purposeful selection of covariates, only two factors were identified as potential predictive covariates: (1) years of education, which significantly predicted observer ratings (β *= -*.33, *p* < .001) and accounted for an additional 12.2% of the variance (*R*^2^ = .122), and (2) years the observer has known an adult who stutters, which approached significance (β *=* .17, *p* = .087), and accounted for an additional 2.8% of the variance (*R*^2^ = .028). After statistically accounting for the contribution of these two observer-based factors, the Post-treatment Video remained a significant, positive predictor of improved observer ratings (β *=* .29, *p* = .004) with the final model accounting for 22.1% of the variance (*R*^2^ = .221; *F*(3, 77) = 7.30, *p* < .001; *f*^*2*^ = .36 [large effect size] see Model 2 in Table 3).

To verify these outcomes, a bootstrapping analysis was conducted to determine 95% confidence interval (CI) for unstandardized beta coefficients of each factor based on 5000 samples. Bootstrap analysis confirmed a significant, positive coefficient for the Post-treatment Video (*p* = .007, [CI: 3.88, 20.72]) while controlling for potential influence of both observer-related factors (years of education: *p* = .003, [CI: -3.99, -.85]; years observers have known an adult who stutters: *p* = .049 [CI: .01, .46], see Table 3).

### Ancillary analyses

The Pre- and Post-treatment Video stimuli included stuttering severity that was rated perceptually similar by untrained observers (*n* = 63 Pre-treatment Video, *n* = 57 Post-treatment Video; *p* = .95) and categorically similar according to the SSI-4 scale (Pre-treatment Video: moderate; Post-treatment Video: moderate). Nevertheless, the frequency of stuttering decreased from pre- to post-treatment samples as measured by %SS (10.60% to 8.24%, Δ -22.26%) and by Section 1 of the SSI-4 (7 to 6). Further, a slight decrease was observed in physical concomitants (SSI-4-Section 3: 10 to 9), resulting in a lower Total Score on the SSI-4 for the Post-treatment Video, but the same overall severity classification (moderate, 27 to 25; see Table 1). To determine whether these decreases in frequency and severity in the post-treatment sample impacted observer-ratings of communication competence, a second analysis was conducted with a different adult who stutters wherein an increase in stuttering (i.e., frequency, duration, and physical concomitants), rather than a decrease, was observed pre- to post-treatment.

A detailed description of pre-/post-treatment outcomes, as well as the regression analysis to control for listener-based factors, is provided in S3 Appendix. In summary, findings from the original analyses of RQ1 and RQ2 were replicated. Similar to results of RQ1 of the original analysis, untrained observers (*n* = 96) rated communication competence significantly higher post-treatment (*p* = .040, *d* = .42 [small-to-medium effect size]) for an adult who stutters who demonstrated higher, rather than lower, post-treatment stuttering frequency and severity. Similar to results of RQ2 of the original analysis, post-treatment gains in communication competence were maintained while controlling for potential listener-based factors (*p* = .045, *f*^*2*^ = .24 [medium-large effect size]). These findings, combined with results from the primary analysis, provide counterevidence to the possibility that raters were responding to the interviewee’s stuttering more so than their communication competence.

## Discussion

The primary purpose of this study was to provide social validation of previous clinician- and self-rated gains in communication competence by exploring the perspective of untrained observers. A secondary purpose was to examine whether observer-related demographic factors mediate their perspective. Untrained observers were recruited to view, and then rate, either the Pre- or Post-treatment Video of an adult who stutters who had completed CCT. Similar to clinician- and self-ratings in previous studies, ratings from untrained observers who viewed the Post-treatment Video were higher than the ratings of the untrained observers who viewed the Pre-treatment Video. Two observer-rated factors were identified as significantly associated with communication competency ratings: (1) years of rater education and (2) years the rater had personally known an adult who stutters. Statistically accounting for these factors, the Post-treatment Video ratings remained significantly higher, suggesting that gains in communication competence following CCT are observed by raters unfamiliar with the nature and/or goals of the treatment.

### RQ1: Communication competence gains from the perspective of untrained observers

As described above, untrained observers’ post-treatment ratings were significantly higher than the pre-treatment ratings. These findings are consistent with the significant pre-/post-treatment gains in communication competence reported for adults who stutter via self-ratings [17], and by clinicians [16]. Findings also corroborate significant gains in communication competence observed by clinicians for young children and adolescents [15]. Consistency across ratings of communication competence from three different perspectives–self, clinician, and untrained observers–suggest that the gains reported reflect a meaningful change that is observable beyond the clinical environment.

Unlike previous studies by Byrd and colleagues [15,16], wherein a single clinician rated pre-/post-treatment video samples from multiple adults who stutter (i.e., many-to-one), the structure of observation in the present study was reversed. In those previous studies, analyses captured the variance of treatment outcomes across multiple participants, with rater variance held constant by use of a single clinician rater. In the present study, multiple observers rated communication competence of a single adult who stutters who had completed CCT (i.e., one-to-many) based on viewing only one of the two videos, either the pre-treatment or the post-treatment. Thus, we were able to avoid potential order effects. Moreover, this statistical design allowed us to capture the variance of responses amongst observers from the general public. That being said, it is possible that the gains reported from the perspective of viewing only one participant may or may not be observed when evaluating a group of participants before and after treatment. Although the present study’s data provide preliminary support for the social validity of CCT, a natural next step is to assess the variance of treatment outcomes across multiple participants by a single untrained observer, similar to the one-to-many rating design in Byrd et al. [15,16].

As noted, stuttering frequency and severity were comparable between video samples (Pre-treatment Video, SSI-4: moderate [score 27]; Post-treatment Video, SSI-4: moderate [score 25]), lending support to the notion that ratings of communication competence were not likely influenced by changes in stuttering. It might be suggested that ratings of stuttering severity provided by expert clinicians may not reflect judgments of severity by untrained listeners in the general public. However, the previously described non-significant differences in observer-based ratings of stuttering severity (see Table 1, *p* = .95) between the Pre- and Post-treatment Video samples suggest this was not the case (as also observed for that adult participant rated in S3 Appendix).

Alternatively, it is also possible that the stuttering was perceived as less distracting in the Post-treatment Video because it was accompanied by higher communication competence. For example, Werle et al. [72] reported that untrained observers who viewed a presenter with a stuttering frequency of 15% who demonstrated high communication competence rated their perceptual stuttering as less distracting than observers who viewed the same presenter who exhibited the same 15% stuttering frequency but demonstrated low communication competence.

Further assessment of qualitative feedback from observers in the present study support the findings of Werle et al. [72], and that of Werle and Byrd [61,64], and indicate that fluency was less of a concern in the presence of stronger communication competence in the Post-treatment Video. For example, observers who viewed the Post-treatment Video noted that they certainly heard the interviewee stuttering, but also commented that its importance was offset by communication skills (e.g., “*The interviewee was very articulate and concise in his language and tone of voice*. *He used his hands when speaking*, *which made him appear more animated and that was easier to follow*.*”* “*Even though the interviewee had a stuttering issue*, *he was able to explain himself well*. *He gave good examples when asked for them by the interviewer*.”; “*I believe that [he] communicated well*. *… He looked the interviewer directly in the eyes*, *smiled*, *and nodded*.”*)*. In contrast, observers who viewed the Pre-treatment Video stated that they often focused solely on stuttering (e.g., “*He was not bold and confident about the way he deliver[s] things*. *He is a stammer[er]*”; “*The interviewee has a speech impediment and it is difficult for him to communicate verbally*.”) or stuttering in addition to poor nonverbal communication skills (e.g., “*He seems to stay within his capabilities of communication*, *but his stutter is distracting*. *He answers questions directly*, *but he doesn’t use much eye contact*.*”*; *“He had a stutter and his body language looked tense but he gave great answers*.”)

S2 Appendix provides a full thematic analysis of open-ended text responses provided by untrained observers. In sum, observers who viewed the Post-treatment Video reported more positive comments about stuttering (*n* = 14 comments) and fewer negative comments about stuttering (*n* = 4 comments) than observers who viewed Pre-treatment Video (*n* = 9 positive comments; 36% fewer positive comments; *n* = 19 negative comments; 79% more negative comments). Thus, even though the Pre- and Post-treatment Videos had similar stuttering frequency, and the same severity rating, qualitative data from the present study indicate that, consistent with Werle et al. [72], heightened communication competence of the speaker who received CCT appeared to minimize the potential negative influence of stuttering on observer judgment.

### RQ2: Observer-based factors associated with ratings of communication competence

Two observer demographic factors–years of education and years the observers had known an adult who stutters–were identified as significant predictors of observer-rated communication competence. Specifically, observers rated communication competence to be stronger as the number of years the observer had personally known an adult who stutters increased, but weaker as the amount of education the observer had completed increased. Although observer-rated evaluations of communication competence remained significant upon controlling for these factors during analyses, the potential implications of these two factors warrant discussion.

Regarding the number of years observers have known a person who stutters, previous research has indicated that people who have developed first-hand relationships with any stigmatized groups are likely to report improved overall judgments of persons within that group (e.g., [73,74]), including persons who stutter (e.g., [75,76]; cf. [57,77]). Thus, results of the present as well as past studies strongly suggest that future studies employing observer evaluations of participants who stutter should continue to include and/or control for the length of time participants know someone who stutters. Measurement or statistical control for this variable would be prudent given its long-standing influence on the general public views of individuals who stutter.

There was a significant negative relationship between years of education and communication ratings, wherein observers with higher levels of education often provided lower ratings of the speaker’s communication competence, regardless of the video viewed. To be clear, the mean number of years of education between groups was nearly identical (Pre-treatment Video; *M* = 16.4 years of education; Post-treatment Video, *M* = 16.5 years of education, *p* = .45). This relationship may be similar to well-documented gender and race discrimination in the workplace within the workplace [78–80]. That is, persons of authority hold lower expectations of stereotyped groups, yet also set higher standards for those group members to prove that their competency is equivalent to non-stereotyped groups. If observers with more formal education in the present study also held higher workplace authority, this may have negatively impacted their perception of communication competence. In short, when asked to evaluate the communication of an interviewee who stutters, individuals with more years of education may have provided ratings based on lower expectations, and higher standards, than individuals with fewer years of education.

Negative association between perceived communication competence and years of education is inconsistent with recent research by Werle and Byrd [61,64]. In these studies, findings were taken to suggest a potential positive response bias when raters with higher years of education (i.e., college professors) evaluate students who stutter. Perhaps this is a unique pattern observed for professors and teachers whose job duties require ongoing evaluation of adult students. It is also possible that the dyadic speaking context lowered the likelihood of positive feedback bias found for professors. That is, professors were potentially more likely to overcorrect their personal biases (observed for Werle and Byrd [62,65]) when presented with a speaker in a context for which they regularly provide evaluation (e.g., oral classroom presentations). In that respect, positive response bias for the mock interviews depicted in the present study may have emerged for observers with a history of employment or training in human resources. Taken together, these two factors–years knowing a person who stutters and years of education–significantly influence ratings of communication competence. However, the significance of ratings based on video status (Pre-treatment versus Post-treatment Videos) remained beyond the influence of these two factors. That overall finding, as well as the large number of observer demographic characteristics included in the analyses, provides confidence that the positive effects of CCT are independent from the unique effects of any demographic factor included in this study.

### Limitations and future studies

Although efforts to account for some of the variance were made, we acknowledge that a number of additional factors, known and unknown, beyond the focus of CCT also influence observer judgments. For example, visual nonverbal information (e.g., attire, physical appearance, environments) have a documented effect on evaluator judgments (e.g., [81]). The same adult completed the recordings for both video samples in the present study. The participant was wearing a dress shirt and necktie during the first interview (Pre-treatment Video) but dressed more casually for the post-treatment interview (Post-treatment Video). One might assume that what the participant was wearing in the Pre-treatment Video may be considered more professional attire, and would influence observer ratings, this video was rated less favorably, suggesting that attire was less critical than overall communication competence.

From a methodological standpoint, video samples that naturally vary in length and content also introduce potential confounds. For example, in the present study, the Post-treatment Video was longer and contained more total words than the Pre-treatment Video, introducing the possibility that observers became fatigued or impatient when viewing and prior to VAS rating. However, the Post-treatment Video Sample received a significantly stronger mean rating than the Pre-treatment Video sample, which tempers this concern. It is also possible that, although both mock interviews were unprompted events, and interviewers were unknown to the participants, the participant benefitted from the previous mock interview experience and felt at greater ease in the identical space provided for both interviews. Additional review of communication competence in a different space, or perhaps investigation of content overlap between interviews, would address this point.

Dyadic interviews lend themselves to a specific, one-on-one style of interaction that favors certain adults who stutter more than others, and post-CCT gains may not necessarily generalize to other contexts such as presentations (however, see Coalson et al. [17] for post-CCT gains when aggregated across seven speaking contexts reported by adult participants who stutter). Given that possibility, it is important to note the present study is one entry in a series of clinical studies focused on the outcomes of CCT from a variety of perspectives (e.g., self, clinician, observer). A larger cohort of observers is needed to corroborate preliminary results, and examination of clinical outcomes will continue across multiple contexts, from multiple perspectives, and with multiple measures, in future studies.

From a conceptual standpoint, several factors limit the interpretation of these findings. First, the present authors did not employ a control group (e.g., two videos of an adult who stutters during an interview who did not receive CCT) or a control sample (e.g., a video sample of the same adult who stutters in this study, with stuttering digitally removed). For these reasons, our interpretation of the reported social validation data is restricted to the general public’s opinion of communicative change following CCT. As such, present findings do not inform differences relative to non-stuttering individuals or individuals who have not yet received treatment. Further, the current data are limited to samples recorded pre-treatment and immediately post-treatment, with no long-term follow-up data. Future investigations will prioritize these factors to provide greater context for the social validity data reported in the present study.

An additional limitation is sample size. Prior studies examining pre-/post-treatment rating communication competence consistently yielded “large” or “very large” effect sizes (Byrd et al., 2021, *d-*value range = .75 to 1.18 [6 of 8 competencies]; Byrd et al., 2022: *d*-value range = .86 to 1.30 [8 of 8 competencies]). Therefore, a priori power analysis was based on large effect size (*d* = .80, *n* = 52). It is true that RQ1 with medium-to-large (*d* = .55) effects size yields underpowered results (minimum *n* = 106). However, the findings of RQ1 are well supported by RQ2. Post-hoc power analysis of the linear regression analysis in RQ2 shows that, while also controlling for two other listener-based variables, the partial *R*^*2*^ for the single predictor of Pre-/Post-treatment Video stimuli is more than sufficiently powered (power = .99).

Another consideration is that the explicit instructions provided to untrained observers may have also influenced ratings. Before providing numeric ratings of communication competence, survey observers were provided the following prompt: “Using the scale below, please rate the interviewee’s communication skills.” This phrasing may have biased observers to view the interviewee through the lens of communication, which while important, is not the only factor considered during an interview evaluation. Indeed, a majority of the comments provided by the observers referred to some aspect of fluency or communication (121 of the 152 comments, 79.6%), with smaller proportions referring to personality (24 of 152, or 15.8%) or employability (7 or 152, or 4.6%; see S2 Appendix, Table 3). A more neutral prompt of “Please rate the person’s performance during the interview.” may have limited the potential bias of the observer.

One final consideration is that observers may have been rating communication relative to “effectiveness”, as stated in the prompt, rather than communication “competence,” as referred to by CCT and supporting research. According to Spitzberg [82,83] and Morreale et al. [84], communication competence is a superordinate concept that captures whether a speaker demonstrates communicative behaviors that are both effective *and* appropriate for a given context. Although it is likely that an increase in effectiveness would also result in an increase in competence, differing terminology in survey prompts may yield different observer perceptions and warrants investigation in future studies.

## Conclusion

Findings from the present study provide social validation for the gains in communication competence following CCT that have been previously observed through clinician- and self-ratings. A large cohort of untrained observers rated the Post-treatment Video of an adult who completed CCT higher than the untrained observers who observed the Pre-treatment Video of the same adult. Additional research is needed to obtain ratings of multiple participants who have completed CCT to provide further insight into whether the gains reported by clinicians, and individuals who stutter who complete this treatment, translate meaningfully into the general public’s perception of their communication. However, these data, though preliminary, do demonstrate that communication competence and stuttering are distinct constructs that can be targeted and rated, independently, lending further support for anti-ableist approaches that do not aim to increase fluency or modify stuttered speech.

## Supporting information

S1 Appendix Description of treatment program. Summary of the Blank Center CARE^™^ Model treatment program.
(DOCX)

S2 Appendix Qualitative study. Thematic analysis of free-text responses provided by untrained observers.
(DOCX)

S3 Appendix Replication study. Data replicating original analyses based on a participant with an inverse stuttering frequency and severity profile.
(DOCX)
