# Supplementary material for: Observer-rated outcomes of communication-centered treatment for adults who stutter: A social validation study
Source: PLoS One. 2024 May 16;19(5):e0303024. doi: 10.1371/journal.pone.0303024 (PMC11098369; doi:10.1371/journal.pone.0303024)
Supplement: S1 Appendix — Summary of the Blank Center CARE™ Model treatment program. (DOCX) [file pone.0303024.s001.docx]

**Description of treatment program**

Byrd [1] details the manualized CCT program that serves as one of the four distinct components of the Blank Center CARE^™^ Model (Communication, Advocacy, Resilience, and Education). A brief summary of the 11-week (i.e., 22-session) treatment protocol is provided below. Treatment includes two 60-minute sessions per week consisting of one group session as well as one individual session. Training during the individual sessions provides an opportunity to review what will be covered in group sessions, prepare for the activities, and debrief for the weekly group sessions. During group session, participants work towards strengthening their communication competencies across distinct speaking scenarios, including mock job interviews, small group interactions, impromptu icebreakers, one-on-one interactions with unfamiliar persons, and multiple presentations that vary both in purpose (e.g., informative, persuasive, inspirational) and audience composition (e.g., small and large groups, familiar and unfamiliar listeners).

● **Week 1** (Sessions 1 and 2): Participants are introduced to the competencies that comprise effective communication (i.e., language use, language organization, speech rate, intonation, volume, gestures, body position, eye contact, facial affect). Participants receive focused training in effective **body positioning and gestures**. Participants complete self-ratings of these communication competencies following an impromptu small group presentation to unfamiliar persons.

● **Week 2** (Sessions 3 and 4): Participants identify and describe core components of communication competence and receive focused training in effective use of **facial affect**, while simultaneously continuing to strengthen competencies addressed in the prior sessions. Participants complete self-ratings of communication competencies following a small group informative speech presentation to unfamiliar persons.

● **Week 3** (Sessions 5 and 6): Participants identify and describe core components of communication competence and receive focused training in **turn-taking and listener awareness**, while simultaneously continuing to strengthen competencies addressed in prior sessions. Participants complete self-ratings of their communication competencies following impromptu dyadic exchanges as well as impromptu small group presentations to unfamiliar persons.

● **Week 4** (Sessions 7 and 8): Participants identify and describe core components of communication competence and receive focused training in effective **vocal variety (i.e., volume, rate, intonation)**, while simultaneously continuing to strengthen competencies addressed in prior sessions. Participants complete self-ratings of communication competencies following impromptu dyadic exchanges, as well as impromptu small group presentations to unfamiliar persons.

● **Week 5** (Sessions 9 and 10): Participants identify and describe core components of communication competence and receive focused training in effective **language use and organization**, while simultaneously continuing to strengthen competencies addressed in prior sessions. Participants complete self-ratings of communication competencies after completing 10+ dyadic interactions with unfamiliar persons.

● **Week 6** (Sessions 11 and 12): Participants identify and describe core components of communication competence and receive additional focused training in **stuttering openly** - that is, making no attempts to avoid stuttering, increase fluency, and/or modify moments of stuttering. Participants complete self-ratings of communication competencies following an open mic presentation to unfamiliar persons in a public forum.

● **Week 7** (Sessions 13 and 14): Participants **review and practice all core components of communication competence simultaneously**. Participants complete self-ratings of communication competencies following a persuasive speech given to a small group and a large group of unfamiliar persons.

● **Week 8** (Sessions 15 and 16): Participants **review and practice all core components of communication competence**. Participants complete self-ratings of communication competencies after serving as an interviewee in a series of panel interviews, with potential employers across diverse professions.

● **Week 9** (Sessions 17 and 18): Participants **review and practice all core components of communication competence**. Participants complete self-ratings of communication competencies following impromptu dyadic exchanges as well as impromptu presentations to a small group of unfamiliar persons.

● **Week 10** (Sessions 19 and 20): Participants **review and practice all core components of communication competence**. Participants complete self-ratings of communication competencies following impromptu dyadic exchanges as well as impromptu presentations to a small group of unfamiliar persons.

● **Week 11** (Sessions 21 and 22): Participants **review and practice all core components of communication competence**. Participants complete a self-rating of communication competencies after providing a prepared presentation to a small group and a large group of unfamiliar persons.

1. Byrd, C. Blank Center CARE^™^ Model: Application for school-age children who stutter. 2023. Dream. Speak. Live. Publications. Available at <https://blankcenterforstuttering.org/blank-center-care-model-manual/>
