# Supplementary material for: Observer-rated outcomes of communication-centered treatment for adults who stutter: A social validation study
Source: PLoS One. 2024 May 16;19(5):e0303024. doi: 10.1371/journal.pone.0303024 (PMC11098369; doi:10.1371/journal.pone.0303024)
Supplement: S2 Appendix — Thematic analysis of free-text responses provided by untrained observers. (DOCX) [file pone.0303024.s002.docx]

**Thematic analysis**

Quantitative outcomes reported for the primary analysis suggest that changes in communication competence, as measured by 100-point VAS rating scale, were perceived by untrained observers post-treatment. Observers were also asked to provide a free-text response to the following question: “What influenced your rating of the interviewee's communication skills? Please describe any and all behaviors, reactions, and/or messages that contributed to your rating.” To examine what influenced observers’ ratings, a thematic analysis [1] was conducted using the free-text written responses provided by the 81 observers who participated.

The thematic analysis was conducted with four predetermined primary themes: (1) fluency, (2) communication, (3) personality, and (4) employability. Each primary theme was either central to the purpose of this manuscript (communication, fluency) or dominant within research that examines third-party perception of individuals who stutter (personality [2-6]; employability [7-9]). The theme of Communication was subdivided into subthemes that correspond with the targeted communication competencies of the Blank Center CARE^™^ Model (as described in Byrd [10]; also similar to rubric used in Byrd et al. [11] and Coalson et al. [12]), as well as Spitzberg’s [13-14] taxonomy of communication competence and the description of communication competence developed by the National Communication Association [15-16]: (1) language (use, organization), (2) voice (pitch, rate, volume), (3) gestures, (4) body, (5) eye contact, and (6) affect. A subtheme termed Overall was also included under the Communication theme to account for comments that describe the interviewee’s communication without reference to specific aspect(s) of communication. Finally, each comment that corresponded with primary theme and subtheme was coded for valence (Positive, Negative, Mixed, and Neutral). Free-text responses were segmented into meaning units, and meaning units were coded so that each unit received only one code that reflected both valence and primary theme (e.g., positive + communication, negative + personality, mixed + fluency, neutral + employability, etc.) or, in the case of Communication, valence and subtheme (e.g., positive + language, negative + gestures, mixed + voice, etc.). Table 1 provides a detailed summary and definition of each primary theme (Fluency, Communication, Personality, Employability) and subthemes.

**Table 1. Descriptions of Themes and Subthemes.**

| **Theme** | **Description** |
| --- | --- |
| 1. **Fluency** | **Fluency is defined as comments that refer to the speaker's stuttering, stammering, or (dis)fluency during interaction.** |
| Positive | Positive evaluation of speaker's fluency. This includes comments wherein the presence of stuttering was acknowledged and then accompanied by favorable evaluation of communication and/or speaker. |
| Negative | Negative evaluation of speaker's fluency. This includes comments wherein the presence of stuttering was acknowledged and then accompanied by unfavorable evaluation of communication and/or speaker. |
| Mixed | Mixed evaluation of speaker's fluency. |
| Neutral | Acknowledgment of speaker's fluency without positive or negative evaluation. |
|  |  |
| 1. **Communication** | **Communication is defined as comments that refer to the speaker's overall communication, or specific communication competencies targeted during treatment (i.e., language use and organization, speech rate, intonation, volume, gestures, body position, eye contact, facial affect).** |
| *Overall* | *Overall refers to the speaker's general communication.* |
| Positive | Positive evaluation of speaker's overall communication competencies. |
| Negative | Negative evaluation of speaker's overall communication competencies. |
| Mixed | Mixed evaluation of speaker's overall communication competencies. |
| Neutral | Acknowledgment of speaker's overall communication without positive or negative evaluation. |
| *Language* | *Language refers to the appropriate use of language during interaction (e.g., expected opening closing remarks? appropriately formal vocabulary?) or organization of language (e.g., open with small talk? on-topic or wandering? succinct and clear?).* |
| Positive | Positive evaluation of speaker's language use or organization. |
| Negative | Negative evaluation of speaker's language use or organization. |
| Mixed | Mixed evaluation of speaker's language use or organization. |
| Neutral | Acknowledgment of speaker's language use or organization without positive or negative evaluation. |
| *Voice* | *Voice refers to appropriate intonation (e.g., pitch varied for emphasis?), volume (e.g., varied for emphasis and strong enough to be heard?), or rate (e.g., speed easily understood and varied for emphasis?) during interaction. Pitch, rate, and volume in this context refer to pragmatic use rather than an effort to support fluent speech.* |
| Positive | Positive evaluation of speaker's rate, pitch, or volume. |
| Negative | Negative evaluation of speaker's rate, pitch, or volume. |
| Mixed | Mixed evaluation of speaker's speech rate, pitch, or volume. |
| Neutral | Acknowledgment of speaker's rate, pitch, or volume without positive or negative evaluation. |
| *Gestures* | *Gestures refers to appropriate movement of hands and arms during interaction (e.g., used to emphasize message? visible? arms not crossed? not gripped, rubbing legs, or in pockets?).* |
| Positive | Positive evaluation of speaker's gestures. |
| Negative | Negative evaluation of speaker's gestures. |
| Mixed | Mixed evaluation of speaker's gestures. |
| Neutral | Acknowledgment of speaker's gestures without positive or negative evaluation. |
| *Body* | *Body refers to speaker's appropriate body positioning and movement for speaking context (e.g., open and confident posture? 'owned the space'? distracting body movements?).* |
| Positive | Positive evaluation of speaker's body position. |
| Negative | Negative evaluation of speaker's body position. |
| Mixed | Mixed evaluation of speaker's body position. |
| Neutral | Acknowledgment of speaker's body position without positive or negative evaluation. |
| *Eye Contact* | *Eye contact refers to speaker using appropriate eye contact with listener (e.g., looked at listener and not avoided? eye contact varied?).* |
| Positive | Positive evaluation of speaker's eye contact. |
| Negative | Negative evaluation of speaker's eye contact. |
| Mixed | Mixed evaluation of speaker's eye contact. |
| Neutral | Acknowledgment of speaker's eye contact without positive or negative evaluation. |
| *Affect* | *Affect refers to whether the speaker's facial affect was appropriate for the speaking context (e.g., smiled occasionally to demonstrate enthusiasm or interest? expression varied to demonstrate enthusiasm or interest? visibly uncomfortable during interaction?).* |
| Positive | Positive evaluation of speaker's facial affect. |
| Negative | Negative evaluation of speaker's facial affect. |
| Mixed | Mixed evaluation of speaker's facial affect. |
| Neutral | Acknowledgment of speaker's facial affect without positive or negative evaluation. |
|  |  |
| 1. **Personality** | **Personality is defined as comments that reference specific personality traits attributed to the speaker.** |
| Positive | Positive evaluation of speaker's personality or attribution of positive personality trait(s). |
| Negative | Negative evaluation of speaker's personality or attribution of negative personality trait(s). |
| Mixed | Mixed evaluation of speaker's personality or specific personality trait(s). |
| Neutral | Acknowledgment of overall personality or personality trait(s) without positive or negative evaluation. |
|  |  |
| 1. **Employability** | **Employability is defined as comments that refer to the likelihood of hiring the speaker after the interview.** |
| Positive | Positive evaluation of willingness to hire interviewee. |
| Negative | Negative evaluation of willingness to hire interviewee. |
| Mixed | Mixed evaluation of willingness to hire interviewee. |
| Neutral | Acknowledgment of employability without positive or negative evaluation. |

From the 81 observers, a total of 152 meaningful comments were coded (*n* = 76 Pre-treatment Video, *n* = 76 Post-treatment Video). Table 2 provides example quotes for each primary and subtheme, if provided, by an observer.

**Table 2. Example Statements by Theme and Subtheme.**

| **Theme** | **Example** |
| --- | --- |
| 1. **Fluency** |  |
| Positive | *Even though he has difficulties in speaking he communicated well.*  *I believe that he had very good communication skills - despite the occasional stutter. He was able to answer all the questions effectively.*  *He stuttered, but he knew exactly what he was talking about and what he wanted to stay. The stuttering didn't really take away from that.* |
| Negative | *He was not bold and confident about the way he deliver[s] things. He is a stammer[er].*  *I think it is really not easy for interviewee to communicate to other people with the way how he speaks.* |
| Mixed | *He was rather hesitant in his answers. Seemed to stumble in the beginning and then gained control of his thoughts.* |
| Neutral | *The interviewee stutters a lot.* |
|  |  |
| 1. **Communication** |  |
| *Overall* |  |
| Positive | *[He] was attentive and responsive to the interviewer, and answered all the questions directly.*  *He seemed to be a relaxed person who communicates well.*  *His responses are on-point.* |
| Negative | *The interviewee's communication skill is not effective.*  *… [his] lacking of communication skill.* |
| Mixed | *The interviewee communication skills is somewhat okay.* |
| Neutral | N/A |
| *Language* |  |
| Positive | *[He] also gave some thought to answers before he just blurted something out.*  *The interviewee provided thoughtful answers to the questions that were being asked.* |
| Negative | *[The] interviewee was vague many times.*  *The interviewee tended to be very wordy in his responses and not get to the point or provide much detail to the answers.* |
| Mixed | *Good language but he could not communicate it properly.* |
| Neutral | N/A |
| *Voice* |  |
| Positive | *He had a nice tenor to his voice**.* |
| Negative | N/A |
| Mixed | *He was a bit slower in phrasing his sentences, but eventually got the point across.* |
| Neutral | N/A |
| *Gestures* |  |
| Positive | *He used his hands when speaking, which made him appear more animated and that was easier to follow.* |
| Negative | *He also had a nervous habit of grabbing at his tie that was becoming distracting and interfering with my ability to concentrate on his answers.* |
| Mixed | N/A |
| Neutral | *He used his hands quite a bit.* |
| *Body* |  |
| Positive | *His bodily communication also helped a lot.* |
| Negative | *He didn’t maintain the correct body language.* |
| Mixed | N/A |
| Neutral | N/A |
| *Eye Contact* |  |
| Positive | *He did maintain eye contact and was very confident interpersonally.* |
| Negative | *I noticed his eye contact with the interviewer was, for the most part, lacking.* |
| Mixed | N/A |
| Neutral | N/A |
| *Affect* |  |
| Positive | *He is very friendly and social from his smile.* |
| Negative | N/A |
| Mixed | N/A |
| Neutral | N/A |
|  |  |
| 1. **Personality** |  |
| Positive | *He maintains a very pleasant demeanour and is obviously bright.* |
| Negative | *He was nervous about the interview.* |
| Mixed | *A bit nervous at times but still coming through with a good answer.* |
| Neutral | N/A |
|  |  |
| 1. **Employability** | **Employability is defined as comments that refer to the likelihood of hiring the speaker after the interview.** |
| Positive | *I would have hired him.* |
| Negative | *[Stuttering] is not his fault, but it would be a factor that employers will consider.* |
| Mixed | N/A |
| Neutral | *Eligible for the interview.* |

*Note.* N/A: No observers provided a response that met the description provided in Table 1

As can been seen in Table 3, comments related to fluency were more positive for those observers who viewed the Post-treatment Video (14 positive comments) compared to observers who viewed the Pre-treatment Video (9 positive comments), resulting in 36% increase in positive comments from untrained observers for speaker who had completed treatment. Comments referring to fluency were also less negative for the Post-treatment Video (4 negative comments) compared to the Pre-treatment Video (19 negative comments), resulting in a 79% decrease in negative comments from untrained observers for the speaker who had completed treatment.

**Table 3. Summary of Qualitative Analysis by Theme and Subtheme.**

|  | N | Pre-Tx | Post-Tx | % change |
| --- | --- | --- | --- | --- |
| Total | 152 | 76 | 76 | 0% |
|  |  |  |  |  |
| **1. Fluency** | **52** | **30** | **22** | **-27%** |
| Positive | 23 | 9 | 14 | +36% |
| Negative | 23 | 19 | 4 | -79% |
| Neutral | 3 | 1 | 2 | +50% |
| Mixed | 3 | 1 | 2 | +50% |
|  |  |  |  |  |
| **2. Communication** | **69** | **29** | **40** | **+28%** |
|  |  |  |  |  |
| ***Overall*** | ***18*** | ***8*** | ***10*** | +20% |
| Positive | 9 | 3 | 6 | +50% |
| Negative | 4 | 4 | 0 | -100% |
| Neutral | – | – | – | – |
| Mixed | 5 | 1 | 4 | +75% |
|  |  |  |  |  |
| ***Language*** | **25** | **10** | **15** | **+33%** |
| Positive | 15 | 6 | 9 | +33% |
| Negative | 6 | 2 | 4 | +100% |
| Neutral | – | – | – | – |
| Mixed | 4 | 2 | 2 | 0% |
|  |  |  |  |  |
| ***Voice*** | **4** | **3** | **1** | **-67%** |
| Positive | 3 | 2 | 1 | -50% |
| Negative | – | – | – | – |
| Neutral | – | – | – | – |
| Mixed | 1 | 1 | 0 | -100% |
|  |  |  |  |  |
| ***Gestures*** | **5** | **2** | **3** | **+33%** |
| Positive | 2 | 0 | 2 | +100% |
| Negative | 2 | 2 | 0 | -100% |
| Neutral | 1 | 0 | 1 | +100% |
| Mixed | - | - | - | - |
|  |  |  |  |  |
| ***Body*** | **7** | **2** | **5** | +60% |
| Positive | 3 | 0 | 3 | +100% |
| Negative | 4 | 2 | 2 | 0% |
| Neutral | – | – | – | – |
| Mixed | – | – | – | – |
|  |  |  |  |  |
| ***Eye Contact*** | **8** | **3** | **5** | **+40%** |
| Positive | 5 | 2 | 3 | +33% |
| Negative | 3 | 1 | 2 | +50% |
| Neutral | – | – | – | – |
| Mixed | – | – | – | – |
|  |  |  |  |  |
| ***Affect*** | **2** | **1** | **1** | **0%** |
| Positive | 2 | 1 | 1 | 0% |
| Negative | – | – | – | – |
| Neutral | – | – | – | – |
| Mixed | – | – | – | – |
|  |  |  |  |  |
| **3. Personality** | **24** | **14** | **10** | -29% |
| Positive | 12 | 6 | 6 | 0% |
| Negative | 7 | 5 | 2 | -60% |
| Neutral | – | – | – | – |
| Mixed | 5 | 3 | 2 | -33% |
|  |  |  |  |  |
| **4. Employability** | **7** | **3** | **4** | **+25%** |
| Positive | 5 | 1 | 4 | +75% |
| Negative | 1 | 1 | 0 | -100% |
| Neutral | 1 | 1 | 0 | -100% |
| Mixed | – | – | – | – |
| *Note.* Pre-Tx: Pre-treatment Video; Post-Tx: Post-treatment Video | | | | |

**References**

1. Crabtree, BF, Miller, WL. A template approach to text analysis: Developing and using codebooks. In: Crabtree, L Miller, WL, editors. Doing Qualitative Research. Newbury Park, CA: Sage; 1992. P. 93–109.
2. Schloss PJ, Espin CA, Smith MA, Suffolk DR. Developing assertiveness during employment interviews with young adults who stutter. J Speech Hear Disord. 1987a Feb;52(1):30-36. Doi: 10.1044/jshd.5201.30. PMID: 3807342.
3. Schloss PH, Freeman, CA, Smith, MA, Espin, CA. Influence of assertiveness training on the stuttering rates exhibited by three young adults. J Fluency Disord. 1987b Oct;12(5):333-353. Doi: 10.1016/0094-730X(87)90031-3
4. De Nardo T, Tetnowski JA, Coalson GA. Listener perceptions of stuttering and stuttering modification techniques. J Fluency Disord. 2023 Mar;75:105960. Doi: 10.1016/j.jfludis.2023.105960. Epub 2023 Jan 27. PMID: 36736074.
5. Lass NJ, Ruscello DM, Pannbacker M, Schmitt JF, Middleton GF, Schweppenheiser K. The perceptions of stutterers by people who stutter. Folia Phoniatr Logop. 1995;47(5):247-51. Doi: 10.1159/000266358. PMID: 8563776
6. Werle D, Byrd CT. Professors’ perceptions and evaluations of students who do and do not stutter following oral presentations. Lang Speech Hear Serv Sch. 2022 Jan 5;53(1):133-149. Doi: 10.1044/2021_LSHSS-21-00069. Epub 2021 Dec 3. PMID: 34861764.
7. Gabel RM, Blood GW, Tellis GM, Althouse MT. Measuring role entrapment of people who stutter. J Fluency Disord. 2004;29(1):27-49. Doi: 10.1016/j.jfludis.2003.09.002. PMID: 15026213.
8. Logan KJ, O’Connor EM. Factors affecting occupational advice for speakers who do and do not stutter. J Fluency Disord. 2012 Mar;37(1):25-41. Doi: 10.1016/j.jfludis.2011.11.005. Epub 2011 Nov 23. PMID: 22325920.
9. Klein JF, Hood SB. The impact of stuttering on employment opportunities and job performance. J Fluency Disord. 2004;29(4):255-73. Doi: 10.1016/j.jfludis.2004.08.001. PMID: 15639081.
10. Werle D, Byrd CT. The impact of self-disclosure and strategies for communication competence on professors’ perceptions and evaluations of students who do and do not stutter. J Speech Lang Hear Res. 2022 Sep 12;65(9):3405-3419. Doi: 10.1044/2022_JSLHR-22-00118. Epub 2022 Aug 30. PMID: 36041466.
11. Byrd CT, Coalson GA, Young MM. Targeting communication effectiveness in adults who stutter: A preliminary study. Top Lang Disord. 2022 Jan 1;42(1):76-93.
12. Coalson, GA, Byrd, CT, Werle D., Croft, R, Mahometa, M. Self-perceived communication competence of adults who stutter following communication-centered treatment. Am J Speech Lang Pathol. Forthcoming. 2024.
13. Spitzberg BH, Cupach WR. Interpersonal skills. In: Knapp ML & Daly JA, editors. The Sage handbook of interpersonal communication. 4^th^ ed. Los Angeles (CA); SAGE; 2011. P. 481-524.
14. Spitzberg BH. (Re)Introducing communication competence to the health professions. J Public Health Res. 2013 Dec 1;2(3):e23. Doi: 10.4081/jphr.2013.e23. PMID: 25170494; PMCID: PMC4147740.
15. Spitzberg BH. The conversational skills rating scale: An instructional assessment of interpersonal competence. National Communication Association; 2007.
16. Morreale S, Moore M, Surges-Tatum D, Webster L. The competent speaker speech evaluation form – Second edition. National Communication Association; 2007.
