# Supplementary material for: Observer-rated outcomes of communication-centered treatment for adults who stutter: A social validation study
Source: PLoS One. 2024 May 16;19(5):e0303024. doi: 10.1371/journal.pone.0303024 (PMC11098369; doi:10.1371/journal.pone.0303024)
Supplement: S3 Appendix — Data replicating original analyses based on a participant with an inverse stuttering frequency and severity profile. (DOCX) [file pone.0303024.s003.docx]

**Replication**

To replicate findings with an adult who stutters with an inverse frequency and severity profile, a participant was selected as interviewee that demonstrated, as closely as possible, an *increase* in stuttering post-treatment that was comparable to the decrease observed post-treatment for the interviewee in the original analysis. The interviewee was an 18-year-old White monolingual female who stutters. As seen in Table 1, stuttering increased from Pre- to Post-treatment Video stimuli 38.7% as measured by SS% (5.5% to 8.9%, respectively), which corresponded with an increased score for frequency subscale on the Stuttering Severity Insturiment-4 [1], Section 1 (10 to 14). The participant also demonstrated increased stuttering duration (SSI-4, Section 2: 6 to 10) and physical concomitants (SSI-4, Section 3: 6 to 7), resulting in an overall increase in SSI-4 Total (22 to 31) and an increased SSI-4 severity classification pre- to post-treatment (Mild to Moderate). Similar to the original analysis, a group of untrained observers rated stuttering severity on a 100-point visual analog scale (VAS) for the Pre-treatment Video (*N* = 42) and the Post-treatment Video (*N* = 43). No significant difference was observed between samples (*p* = .65, see Table 1).

**Table 1. Characteristics of Pre-Treatment and Post-Treatment Video Stimuli.**

|  | Pre-treatment Video | Post-treatment Video |
| --- | --- | --- |
| %SS^a^ | 5.47% | 8.93% |
| Total words | 603 | 549 |
| SSI-4^b^ | Mild | Moderate |
| Frequency | 10 | 14 |
| Duration | 6 | 10 |
| Physical Concomitants | 6 | 7 |
| Total | 22 | 31 |
| Observer-Rated Severity (100-point VAS) |  |  |
| *M* (SE) | 27.19 (3.04) | 29.07 (2.75) |
| *N* | 42 | 43 |
| Length of Video | 6 min, 5 sec | 5 min, 26 sec |

^a^Percent of stuttered speech, disfluency types based on Yairi and Ambrose [2]

^b^Stuttering Severity Instrument-4^th^ Edition [1]

**Survey administration and observer description**

Survey description and administration were identical to the original analysis. A separate, non-overlapping group of 105 untrained observers watched one of two possible videos (*n* = 53 Pre-treatment Video, *n* = 52 Post-treatment Video). Of these 105, eight were excluded as they identified as an adult who stutters (*n* = 49 Pre-treatment Video, *n* = 48 Post-treatment Video). Of the remaining 97, one observer failed to pass an attention check question and was removed. The final cohort was comprised of 96 untrained observers (*n* = 48 Pre-treatment Video, *n* = 48 Post-treatment Video). Table 2 provides demographic characteristics of untrained observers.

**Table 2. Demographic Characteristics of Untrained Observer Groups.**

|  | Video Stimuli | |  |
| --- | --- | --- | --- |
|  | Pre-treatment | Post-treatment | *N* |
| *N* | 48 | 48 | 96 |
| Age | 38.8 (12.4) | 41.8 (14.6) | 45.0 (9.1) |
| Race |  |  |  |
| Native American or Alaskan Native | 0 | 0 | 0 |
| Asian | 6 | 6 | 12 |
| Black or African American | 5 | 3 | 8 |
| Native Hawaiian or Pacific Islander | 0 | 0 | 0 |
| White | 35 | 37 | 72 |
| Other Identification | 2 | 2 | 4 |
| Ethnicity |  |  |  |
| Not Hispanic or Latino | 47 | 48 | 95 |
| Hispanic or Latino | 1 | 0 | 1 |
| Self-Identified Gender |  |  |  |
| Male | 23 | 21 | 44 |
| Female | 23 | 27 | 50 |
| Other Identification | 2 | 0 | 2 |
| Years of Education | 16.8 (3.2) | 16.0 (3.2) | 16.4 (3.2) |
| Primary Language |  |  |  |
| Arabic | 0 | 1 | 1 |
| Cantonese | 1 | 0 | 1 |
| English | 43 | 45 | 88 |
| French | 1 | 0 | 1 |
| Korean | 1 | 1 | 2 |
| Tamil | 0 | 1 | 1 |
| Turkish | 1 | 0 | 1 |
| Urdu | 1 | 0 | 1 |
| Knows adult who stutters | 12 | 13 | 25 |
| Years known | 3.6 (8.8) | 3.9 (9.4) | 3.7 (9.1) |
| Invisible or mixed disability | 10 | 12 | 22 |

*Note.* Means and standard deviations (in parenthesis) reported for age, years of education, and years known.

**Analyses**

Analyses of communication competence were identical to RQ1 and RQ2 in the original analysis. First, an independent, two-tailed *t*-test (α = .05) was conducted to compare untrained observers’ evaluation of communication competency depicted in one of two stimuli – the Pre-treatment Video or the Post-treatment Video. Video type served as the independent variable, and communication competence ratings from the 100-point VAS of communication competence served as the dependent variable. Findings were verified by non-parametric analysis (Mann-Whitney *U*, α = .05).

To assess which observer-rated variables, if any, held meaningful predictive value of communication competence ratings, we again applied Hosmer et al.’s [3] method for purposeful selection of covariates described in the original analysis. To maintain relatively even distribution amongst demographic categories, similar to the original analysis, non-binary categorical variables with infrequent responses, such as primary language other than English (*n* = 8) and self-identified race (Asian, *n* = 12; Black or African American, *n* = 8; Race not Described, *n* = 4), were transformed to create a binary variable when possible. In the case of the two observers who self-identified as non-binary gender, each observer was randomly assigned to one of the two gender categories (i.e., male/female). Due to disproportionately high self-identification as Not Hispanic or Latino (*n* = 95) rather than Hispanic/Latino (*n* = 1), ethnicity was removed as a potential predictor variable. All main effects, as well as any significant interaction terms, remaining after Hosmer et al.’s method are reported in Table 3, along with additional bootstrap analysis (95% confidence intervals; 5000 samples).

**Results**

Findings indicate that observers perceived significantly stronger communication competence when viewing the video of a speaker post-treatment (Post-treatment Video; *M* = 66.1, *SD* = 20.3) than when viewing a video of a speaker pre-treatment (Pre-treatment Video; *M* = 57.2, *SD* = 21.8), *t*(94) = 2.08, *p* = .040, *d* = .42 (small-to-medium effect size, Cohen [4]). Findings were confirmed via nonparametric analysis, *U*(48, 48) = 868.50, *z* = 2.08, *p* = .037. Findings replicate the original analysis.

A linear regression was conducted to determine the contribution of eight observer-related factors (i.e., age, race, gender, years of education, non-English primary language, knowing an adult who stutters, number of years the observer has known an adult who stutters, invisible diagnosis) upon ratings of communication competence for the adult who stutters. As expected, the video stimuli depicting the adult speaker post-treatment was a significant predictor of higher observer ratings when entered as the lone predictor variable (β = .21, *p* = .040), explaining 4.4% of the variance *F*(1, 94) = 6.03, *p* = .040 (see Model 1 in Table 3). Upon completing Hosmer et al.’s [3] purposeful selection of covariates, only two factors were identified as potential predictive covariates: (1) primary language other than English, which significantly predicted observer ratings (β *= -*.20, *p* = .044) and accounted for an additional 5.3% of the variance, and (2) if the observer personally knew an adult who stutters, which approached significance (β *=* -*.*18*, p* = .078), and accounted for an additional 3.0% of the variance. After accounting for the contribution of these two observer-based factors, viewing the Post-treatment Video remained a significant, positive predictor of improved observer ratings (β *=* .20*, p* = .045, *f^2^* = .24, medium-large effect size; Cohen [4]) with the final model accounting for 12.8% of the variance (*R*^2^ = .128; *F*(3, 92) = 7.30, *p* = .006; see Model 2 in Table 3). Findings replicate the original analysis with respect to video stimuli, albeit with different observer-based predictors included in the model.

To verify these outcomes, a bootstrapping analysis was conducted to determine 95% confidence interval (CI) for unstandardized beta coefficients of each factor based on 5000 samples. Bootstrap analysis confirmed a significant, positive coefficient for video stimuli depicting a post-treatment speaker (*p* = .046, [CI: .29, 16.47]) while controlling for potential influence of both observer-related factors (primary language: *p* = .092, [CI: -35.15, 3.61]; known an adult who stutters: *p* = .099 [CI: -18.26, 1.74], see Table 3).

**Table 3. Summary of Regression Analyses of Stimuli (Pre-treatment Video, Post-treatment Video) and Observer-Based Factors Predicting Communication Competence of an Adult who Stutters, as Rated by Untrained Observers.**

|  | Variable | *B* | 95% CI | *β* | *t* | *p* |  |  | *F* | *df* | *R^2^* |
| --- | --- | --- | --- | --- | --- | --- | --- | --- | --- | --- | --- |
| Model 1 | Intercept | 57.17 | 51.14, 63.20 |  | 18.82 | <.001 |  |  | 4.33 | 1, 94 | .044 |
|  | **Pre-/Post-treatment Video** | **8.94** | **.41, 17.47** | **.21** | **2.08** | **.040** |  |  |  |  |  |
|  |  |  |  |  |  |  |  |  |  |  |  |
| Model 2 | Intercept | 60.92 | 54.54, 67.32 |  | 18.93 | <.001 |  |  | 4.48 | 3, 92 | .128 |
|  | **Pre-/Post-treatment Video** | **8.47** | **-.17, 16.55** | .20 | 2.03 | **.045** |  |  |  |  |  |
|  | Primary language | -15.57 | -30.74, -.41 | -.20 | -2.04 | .044 |  |  |  |  |  |
|  | AWS known | -8.56 | -18.08, .96 | -.18 | 1.79 | .078 |  |  |  |  |  |
|  |  |  |  |  |  |  |  |  |  |  |  |
| Bootstrapped | Intercept |  | 53.89, 67.07 |  |  | <.001 |  |  |  |  |  |
|  | **Pre-/Post-treatment Video** |  | **.29, 16.47** |  |  | **.046** |  |  |  |  |  |
|  | Primary language |  | -35.15, 3.61 |  |  | .092 |  |  |  |  |  |
|  | AWS known |  | -18.26, 1.74 |  |  | .099 |  |  |  |  |  |
| *Note.* CI = confidence interval for unstandardized beta coefficients; AWS = adult who stutters | | | | | | | | | | | |

**References**

1. Riley G. Stuttering severity instrument for children and adults – Fourth edition. Pro-Ed; 2009.
2. Yairi E, Ambrose NG. Early childhood stuttering for clinicians by clinicians: Pro-Ed; 2005.
3. Hosmer Jr DW, Lemeshow S, Sturdivant RX. Applied logistic regression. John Wiley & Sons; 2013 Apr 1.
4. Cohen J. Statistical power analysis for the behavioral sciences. Lawrence Erlbaum Associates; 1988.
